# Synthesis and Anti-*Candida* Activity of Cobalt(II) Complexes of Benzene-1,2-Dioxyacetic Acid (bdoaH_2_). X-Ray Crystal Structures of [Co(bdoa)(H_2_O)_3_] ⋅3.5H_2_O and {[CO(phen)_3_](bdoa)}_2_⋅24H_2_O (phen = 1,10-Phenanthroline)

**DOI:** 10.1155/MBD.1999.41

**Published:** 1999

**Authors:** Majella Geraghty, Malachy McCann, Michael Devereux, Fergal Cronin, Martin Curran, Vickie McKee

**Affiliations:** 1 Chemistry Department National University of Ireland Maynooth Co. Kildare Maynooth Ireland; 2 Dublin Institute of Technology Cathal Brugha Street Dublin Ireland; 3 School of Chemistry Queen's University Belfast Ireland

## Abstract

Co(CH_3_CO_2_)_2_⋅4H_2_O reacts with benzene-1,2-dioxyacetic acid (bdoaH_2_) to give the Co_2+_ complexes [Co(bdoa)(H_2_O)_3_]⋅H_2_O (**1a**) and [Co(bdoa)(H_2_O)_3_] ⋅3.5H_2_O (**1b**). Subsequent reaction of **1a** with 1,10- phenanthroline produces [CO(phen)_3_] bdoa⋅10H_2_O (**2a**) and {[CO(phen)_3_](bdoa)}_2_⋅24H_2_O (**2b**). Molecular structures of **1b** and **2b** were determined crystallographically. In **1b** the bdoa^2-^- ligates the metal by two carboxylate oxygens and two ethereal oxygens, whereas in **2b** the bdoa^2-^ is uncoordinated. The Mn^2+^ and Cu^2+^ complexes [Mn(bdoa)(phen)_2_]⋅H_2_O (**3**) and [Cu(pdoa)(imid)_2_] (**4**) were also synthesised, **1a-4** and other metal complexes of bdoa H_2_ (metal = Mn^2+^, Co^2+^ ,Cu^2+^, Cu^+^ ) were screened for their ability to inhibit the growth ofhe yeast *Candida albicans*. Complexes incorporating the 1,10-phenanthroline ligand were the most active.


### SYNTHESIS AND ANTI-*CANDIDA* ACTIVITY OF COBALT(II) COMPLEXES OF BENZENE-1,2-DIOXYACETIC ACID ($bdoaH_2$). X-RAY CRYSTAL STRUCTURES OF $[Co(bdoa)(H_2O)_3]\cdot3.5H_2O$ AND $\{[CO(phen)_3](bdoa)]\}_2\cdot24H_2O$ (phen = 1,10-PHENANTHROLINE)

Majella Geraghty,[1] Malachy McCann,[1] Michael Devereux,[2] Fergal Cronin,[1] Martin Curran[1] and Vickie McKee[3]

[1] Chemistry Department, National University of Ireland Maynooth, Maynooth, Co. Kildare, Ireland
E-mail: mgeraghty@may.ie
[2] Dublin Institute of Technology, Cathal Brugha Street, Dublin, Ireland
[3] School of Chemistry, Queen's University, Belfast, N. Ireland

**Abstract**

$Co(CH_3CO_2)_2\cdot4H_2O$ reacts with benzene-1,2-dioxyacetic acid ($bdoaH_2$) to give the $Co^{2+}$ complexes $[Co(bdoa)(H_2O)_3]\cdot H_2O$ (**1a**) and $[Co(bdoa)(H_2O)_3]\cdot3.5H_2O$ (**1b**). Subsequent reaction of **1a** with 1,10-phenanthroline produces $[Co(phen)_3]bdoa\cdot10H_2O$ (**2a**) and $\{[Co(phen)_3](bdoa)\}_2\cdot24H_2O$ (**2b**). Molecular structures of **1b** and **2b** were determined crystallographically. In **1b** the $bdoa^{2-}$ ligates the metal by two carboxylate oxygens and two ethereal oxygens, whereas in **2b** the $bdoa^{2-}$ is uncoordinated. The $Mn^{2+}$ and $Cu^{2+}$ complexes $[Mn(bdoa)(phen)_2]\cdot H_2O$ (**3**) and $[Cu(bdoa)(imid)_2]$ (**4**) were also synthesised. **1a-4** and other metal complexes of $bdoaH_2$ (metal = $Mn^{2+}$, $Co^{2+}$, $Cu^{2+}$, $Cu^+$) were screened for their ability to inhibit the growth of the yeast *Candida albicans*. Complexes incorporating the 1,10-phenanthroline ligand were the most active.

*Keywords:* crystal structures; benzene-1,2-dioxyacetic acid, 1,10-phenanthroline; metal complexes; *Candida albicans.*

### Introduction

Microbial infections in both humans and animals are diverse in their manifestations, ranging from superficial skin problems, chronic infection of the nails, mouth, throat or vagina to frequently fatal systematic diseases. The persistent use of cytotoxic drugs, corticosteroids, antibiotics and immunosuppressants has resulted in an increase in systemic opportunistic microbial infections. The yeast *Candida albicans,* a commensal of the human body, is considered to be the most important fungal pathogen. Over 75% of women suffer from vaginal candidosis (thrush) at some stage in their lifetime, and systemic candidosis is often fatal in immunocompromised patients.[1-4] The search for novel antifungal drugs has intensified over the last decade and some literature has highlighted the use of metal complexes as useful antifungal agents.[5]

Benzene-1,2-dioxyacetic acid ($bdoaH_2$) contains six potential oxygen donor atoms (four carboxylate oxygens and two ethereal oxygens, Fig. 1) and is an extremely versatile ligand.

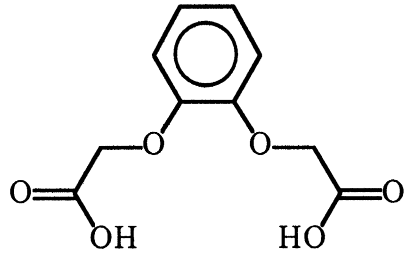

**Figure 1.** Benzene-1,2-dioxyacetic acid ($bdoaH_2$).

To date, $K^+$,[6] $Mg^{2+}$,[7] $Ca^{2+}$,[7] $La^{3+}$,[8] $Mn^{2+}$,[7,9] $Ni^{2+}$,[7] $Cu^{2+}$,[10,11] $Cu^+$ [12] and $Zn^{2+}$ [7] complexes of this aryloxydicarboxylic acid have been structurally characterised. In the present paper we report the full X-ray crystal structures of two cobalt(II) complexes with bdoa$^{2-}$. In addition, the anti-*Candida* activities of bdoaH$_2$ and its $Mn^{2+}$, $Co^{2+}$, $Cu^{2+}$ and $Cu^{+}$ complexes are presented and this, to our knowledge, is the first report of the biological chemistry of both the diacid and its metal complexes.

**Materials and Methods**

Chemicals were purchased from commercial sources and used without further purification. Literature methods were used to prepare [Mn(bdoa)(H$_2$O)$_3$] (**5**),[9] [Cu(bdoa)(H$_2$O)$_2$] (**6**),[11] [Cu$_2$(bdoa)(phen)$_4$]bdoa·13H$_2$O (7) (phen = 1,10-phenanthroline),[11] [Cu$_2$(bdoa)(bipy)$_4$]bdoa·8.66H$_2$O (**8**) (bipy = 2,2'-bipyridine),[10] [Cu(bdoa)(an)$_2$]$_2$an (**9**) (an = aniline),[10] [Cu(bdoa)(NH$_3$)$_2$]·H$_2$O (**10**),[11] [Cu(bdoa)(py)$_2$]·H$_2$O (**11**) (py = pyridine),[11] [Cu(bdoaH)(PPh$_3$)$_3$] (**12**),[12] [Cu(phen)(PPh$_3$)$_2$]bdoaH (**13**).[13] Infrared spectra were recorded as KBr discs in the region 4000-400 cm$^{-1}$ on a Nicolet Impact 400D FT-IR Spectrometer. Magnetic susceptibility measurements were made at room temperature using a Johnson Matthey Magnetic Susceptibility Balance and conductivity measurements were taken at 25 °C using an AGB Scientific Ltd. model 10 conductivity meter. Elemental analysis were carried out by the Microanalytical Laboratory, University College Cork, Ireland. Biological susceptibility testing was conducted using a Labsystems iEMS Reader MF.

**Table 1.** Crystal data.

| | [Co(bdoa)(H$_2$O)$_3$]·3.5H$_2$O (**1b**) | {[Co(phen)$_3$](bdoa)}$_2$·24H$_2$O (**2b**) |
|---|---|---|
| Empirical formula | $C_{10}H_{21}CoO_{12.5}$ | $C_{92}H_{112}Co_2N_{12}O_{36}$ |
| Formula weight | 400.20 | 2079.80 |
| Crystal description | pink plate | orange block |
| Temperature (K) | 153(2) | 153(2) |
| Wavelength (Å) | 0.71073 | 0.71073 |
| Crystal system | monoclinic | monoclinic |
| Space group | C2/c | P2$_1$ |
| a,(Å) | 29.151(4) | 13.123(4) |
| b (Å) | 6.700(1) | 21.166(6) |
| c (Å) | 17.215(2) | 18.809(9) |
| α (°) | 90 | 90 |
| β (°) | 108.74(2) | 110.15(4) |
| γ (°) | 90 | 90 |
| V (Å$^3$) | 3184.0(7) | 4905(3) |
| Z | 8 | 2 |
| Density (calculated, Mg/m$^3$) | 1.670 | 1.408 |
| μ (mm$^{-1}$) | 1.142 | 0.430 |
| F(000) | 1664 | 2180 |
| Crystal size (mm) | 0.52x 0.27 x 0.15 | 0.90 x 0.37 x 0.32 |
| θ range for data collection (°) | 2.0 to 25.0 | 2.0 to 25.0 |
| Index ranges | -1< h < 34 | 0 < h < 14 |
| | -1 < k < 7 | -1 < k < 22 |
| | -20 < l < 19 | -20 < l < 19 |
| Reflections collected | 3359 | 7385 |
| Independent reflections | 2784 ($R_{int}$= 0.087) | 7033 ($R_{int}$ = 0.0526) |
| Data / restraints / parameters | 2784 / 0 / 226 | 7033 / 1 / 914 |
| $T_{max}$, $T_{min}$ (ψ-scans) | 0.954, 0.807 | none |
| Goodness-of-fit on F$^2$ (all data) | 1.058 | 1.089 |
| Final R indices [I >2σ(I)] | R1 = 0.0584 | R1 = 0.0712 |
| | wR2 = 0.1419 | wR2 = 0.2001 |
| R indices (all data) | R1 = 0.0915 | R1 = 0.0949 |
| | wR2 = 0.1648 | wR2 = 0.2285 |
| Largest peak and hole (eA$^{-3}$) | 0.754 and -0.673 | 0.958 and -0.611 |
| Absolute structure parameter | - | 0.04(4) |

Crystal data are summarised in Table 1. Data sets for [Co(bdoa)(H$_2$O)$_3$]·3.5H$_2$O (**1b**) and {[Co(phen)$_3$](bdoa)}$_2$·24H$_2$O (**2b**) were collected on a Siemens P4 four-circle diffractometer and were corrected for Lorentz and polarisation effects, **1b** was also corrected for absorption. Both structures were solved by direct methods (TREF) and refined by full matrix least-squares on $F^2$, using all of the data. All the non-hydrogen atoms were refined with anisotropic atomic displacement parameters; hydrogen atoms bonded to carbon were inserted at calculated positions. In **1b** hydrogen atoms on the full-occupancy water molecules and on O7W were located from difference Fourier maps and not further refined. Hydrogen atoms on the water molecules of **2b** were not included. All programs used in the structure solution and refinement are contained in the SHELXL97 package.[14]

**$[Co(bdoa)(H_2O)_3]\cdot H_2O$ (1a) and $[Co(bdoa)(H_2O)_3]\cdot 3.5H_2O$ (1b).** Complex (**1a**) was prepared using a modification of the literature method.[7] To a solution of $bdoaH_2$ (1.13 g, 5.0 mmol) in water/ethanol (1:4, 150 $cm^3$) was added cobalt(II) acetate tetrahydrate (1.87 g, 7.5 mmol). An immediate color change to deep red was observed and upon stirring the product precipitated as a deep red solid. The solid was filtered off, washed with two portions of ethanol, one portion of diethylether and then dried in air. Yield: 1.46 g (82%). Microanalysis for **1a**: Calc.: C 33.83; H 4.54. Found: C 34.22, H 5.05%. The complex was soluble in water and in ethanol. $\mu_{eff}$ = 5.02 B.M.. $\Lambda_M$ ($H_2O$) = 154 S $cm^2$ $mol^{-1}$. IR: 3387, 2929, 2854, 1567, 1474, 1345, 1314, 1105, 1030, 723, 673, 615, 532 $cm^{-1}$.

Dark pink crystals of $[Co(bdoa)(H_2O)_3]\cdot 3.5H_2O$ (**1b**) deposited overnight from the reaction filtrate and were used for X-ray structure determination.

**$[Co(phen)_3]bdoa\cdot 10H_2O$ (2a) and $\{[Co(phen)_3](bdoa)\}_2\cdot 24H_2O$ (2b).** Complex **1a** (0.57 g, 1.6 mmol) and 1,10-phenanthroline monohydrate (1.6 g, 8.1 mmol) were dissolved in water/ethanol (1:4, 200 $cm^3$) and the deep yellow solution was refluxed for 0.5 h and then concentrated *in vacuo* to *ca* 100 $cm^3$. Orange crystals deposited slowly (21 days) from the solution. Some of the crystals were collected in a small portion of the mother liquor and used for X-ray crystal structure determination where the formulation was shown to be $\{[Co(phen)_3](bdoa)\}_2\cdot 24H_2O$ (**2b**). The remaining crystals **2a** were filtered off, washed twice with a small portion of ethanol and then dried in air. Yield: 0.82 g (51%). Microanalysis for **2a** : Calc.: C 55.04; H 5.22; N 8.37. Found: C 54.50; H 3.38; N 7.91%. Complex **2a** was soluble in water and in ethanol. $\mu_{eff}$ = 4.83 B.M.. $\Lambda_M$ ($H_2O$) = 162 S $cm^2$ $mol^{-1}$. IR: 3387, 2930, 2854, 1567, 1474, 1345, 1313, 1160, 1098, 867, 849, 772, 726, 643 $cm^{-1}$.

**$[Mn(bdoa)(phen)_2]\cdot H_2O$ (3).** To a solution of $[Mn(bdoa)(H_2O)_3]$ (**5**) (0.60 g, 1.80 mmol) in ethanol (80 $cm^3$) was added 1,10-phenanthroline (1.05 g, 5.83 mmol) and the resulting solution was refluxed for 2 h. The precipitated yellow product was filtered off, washed with two small portions of ice-cold ethanol and then dried in air. Yield: 1.10 g (93%). Microanalysis for **3**: Calc.: C 62.10; H 3.99; N 8.52. Found: C 62.00, H 4.30; N 8.13%. The complex was soluble in water and in methanol. $\mu_{eff}$ = 5.76 B.M.. $\Lambda_M$ ($H_2O$) = 54 S $cm^2$ $mol^{-1}$. IR: 1608, 1510, 1462, 1430, 1393, 1344, 1295, 1264, 1240, 1203, 1135, 1043, 913, 870, 849, 818, 778, 760, 700, 636, 587 $cm^{-1}$.

**$[Cu(bdoa)(imid)_2]$ (4).** To a solution of imidazole (imid) (0.92 g, 1.35 mmol) in ethanol (30 $cm^3$) was added $[Cu(bdoa)(H_2O)_2]$ (**6**) (0.20 g, 0.62 mmol). The resulting solution was refluxed for 2 h and then filtered whilst hot. The blue product deposited from the filtrate upon standing for 24 h. The solid product was filtered off and the filtrate allowed to stand. The product was washed with cold ethanol and ether, and then dried in air. On standing for a prolonged period the filtrate yielded *ca.* 0.2 g. of a blue tar. Yield: 0.18 g (68%). Microanalysis for **4**: Calc.: C 45.3; H 3.8; N 13.2. Found: C 43.6; H 3.6; N 13.8%. The complex was soluble in water and in ethanol. $\mu_{eff}$ = 1.82 B.M.. $\Lambda_M$ ($H_2O$) = 105 S $cm^2$ $mol^{-1}$; $\Lambda_M$ (EtOH) = 2 S $cm^2$ $mol^{-1}$. IR: 3130, 1600, 1500, 1410, 1390, 1325, 1255, 1210, 1120, 1070, 1045, 935, 820, 780, 740, 695, 650, 615 $cm^{-1}$.

**Biological studies**

*Candida albicans* (three isolates) were obtained from in St. James's Hospital, Dublin. The isolates were stored on Sabouraud dextrose agar (SDA) plates at 4 °C. Solutions of the test complexes were prepared by dissolving the complex (0.02 g) in distilled water (100 $cm^3$) to yield a stock solution at a concentration of 200 $\mu g$ $cm^{-3}$. The solution was filter sterilised using a Millipore membrane filter (0.45 $\mu m$). Complexes which were insoluble in water were ground to a fine powder and then suspended in sterile distilled water (100 $cm^3$).

**Susceptibility testing method**

RPMI-1640 broth medium (Sigma R 7755) was used for the anti-*Candida* susceptibility testing. The medium (1 $dm^3$) was supplemented with L-glutamine (0.3 g) and morpholinepropanesulfonic acid (MOPS) (34.6 g) and was adjusted to pH 7.0 using sterile NaOH (0.2 M).

The broth microdilution reference method was used.[15] Prior to testing yeast cells were grown on Sabouraud dextrose agar (SDA) at 37 °C for 24 h. Cell suspensions were prepared in sterile phosphate buffered saline (5 $cm^3$) to a density of 0.5 McFarland standard. A 1:100 dilution of the 0.5 McFarland standard yeast suspension was made in RPMI-1640 medium. The cell concentration of the final inoculum was 3.5 x $10^4$ - 5.0 x $10^5$ cells $cm^{-3}$. The prepared cell suspension (90 µl) was dispensed into microtitre plates and to this was added the test stock complex solution (10 µl) to yield working solutions of the test complexes of concentration 20 µg $cm^{-3}$. Plates were then incubated for 24 h at 37 °C with continuous shaking. Each complex was assessed in triplicate and three independent experiments were performed. The resulting nine data points were statistically analysed using ANOVA one-way analysis of variance followed by Tukey's family error rate.

**Results and Discussion**

The pink complex [Co(bdoa)$(H_2O)_3$]·$H_2O$ (**1a**) was prepared in high yield by reacting cobalt(II) acetate tetrahydrate with bdoa$H_2$, and crystals of composition [Co(bdoa)$(H_2O)_3$]·3.5$H_2O$ (**1b**) were isolated from the reaction filtrate. The X-ray crystal structure of **1b** was determined (Fig. 2, Table 2)[16] and the cobalt(II) ion is seven-coordinate, being bonded to three water molecules, a carboxylate oxygen from each end of the bdoa$^{2-}$ and, much more weakly, to the two ether oxygens. The [Co(bdoa)$(H_2O)_3$] unit of **1b** is isostructural with the manganese(II) species [Mn(bdoa)$(H_2O)_3$] (**5**).[9]

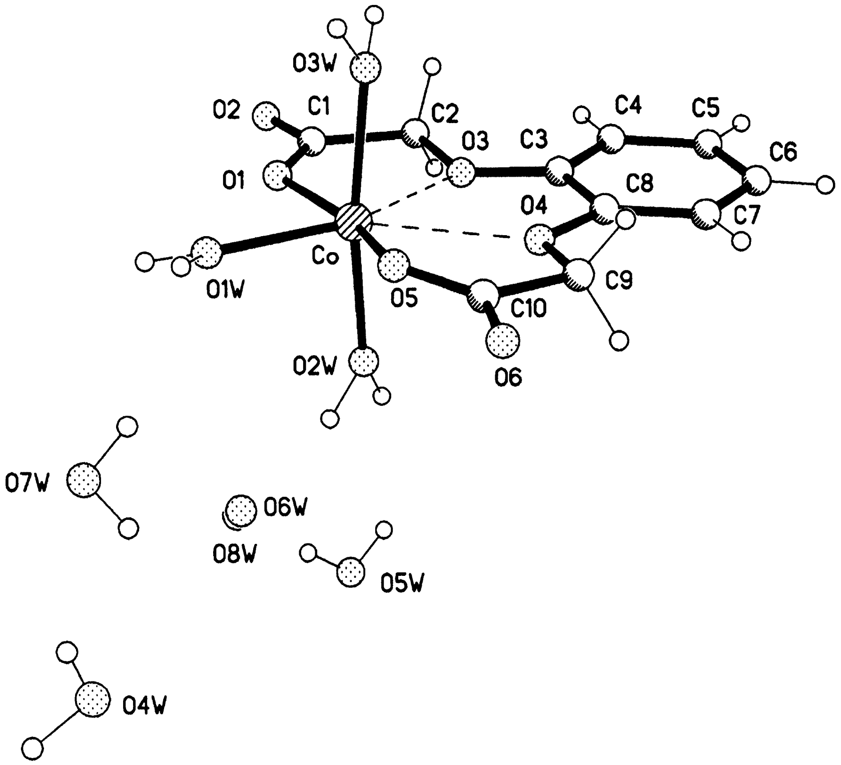

**Figure 2.** X-ray crystal structure of [Co(bdoa)$(H_2O)_3$]·3.5$H_2O$ **1b**

Reaction of **1a** with 1,10-phenanthroline (1:5 mol ratio) gave the tris(phenanthroline) adduct [Co(phen)$_3$]bdoa·10$H_2O$ (**2a**), and crystals of composition {[Co(phen)$_3$](bdoa)}$_2$·24$H_2O$ (**2b**) were isolated from the mother liquor. The X-ray crystal structure of **2b** (Fig. 3 and 4, Table 3) shows that the bdoa$^{2-}$ and the three water ligands have been displaced from the coordination sphere of the metal in **1b** and replaced by three chelating phenanthrolines. The bdoa$^{2-}$ simply functions as a counter dianion. The asymmetric unit of **2b** contains two independent (but similar) [Co(phen)$_3$]$^{2+}$ cations, two independent (and different) bdoa$^{2-}$ anions and twenty four water molecules. There is nothing remarkable in the geometry of the [Co(phen)$_3$]$^{2+}$ cations, although they interact with each other by π–π stacking, forming chains along the *a* direction (interplanar distance between the N1C-C12C ring, coordinated to Co1, and the N1D-C12D ring, coordinated to Co2, is ~3.5 Å). The two bdoa$^{2-}$ anions differ in that the first is approximately coplanar while, in the second, one of the carboxylate groups is almost perpendicular to the phenyl ring. This is likely to be a consequence of the very extensive hydrogen-bonding network which links the anions and all twenty four water molecules into a three-dimensional grid with spaces for the cation chains.

**Table 2.** Selected bond lengths [Å] and angles [°] for $[Co(bdoa)(H_2O)_3]\cdot 3.5H_2O$ (**1b**).

| | | | |
|---|---|---|---|
| Co-O(1) | 2.125(4) | Co-O(5) | 2.117(4) |
| Co-O(3) | 2.440(4) | Co-O(4) | 2.509(4) |
| Co-O(1W) | 2.084(4) | Co-O(2W) | 2.028(5) |
| Co-O(3W) | 2.051(4) | | |
| O(1)-Co-O(3) | 68.71(15) | O(5)-Co-O(4) | 67.04(15) |
| O(1W)-Co-O(1) | 83.06(18) | O(1W)-Co-O(5) | 80.94(18) |
| O(2W)-Co-O(1) | 90.1(2) | O(2W)-Co-O(5) | 95.9(2) |
| O(3W)-Co-O( 1) | 87.20(18) | O(3W)-Co-O(5) | 89.77(19) |
| O(1W)-Co-O(3) | 151.66(17) | O(1W)-Co-O(4) | 146.89(17) |
| O(2W)-Co-O(3) | 86.82(18) | O(2W)-Co-O(4) | 84.5(2) |
| O(3W)-Co-O(3) | 81.88(17) | O(3W)-Co-O(4) | 88.65(18) |
| O(1)-Co-O(4) | 129.59(15) | O(5)-Co-O(3) | 127.40(16) |
| O(5)-Co-O(1) | 162.95(17) | O(3)-Co-O(4) | 60.96(14) |
| O(2W)-Co-O(1W) | 90.9(2) | O(3W)-Co-O(1W) | 99.80(19) |
| O(2W)-Co-O(3W) | 168.59(19) | | |

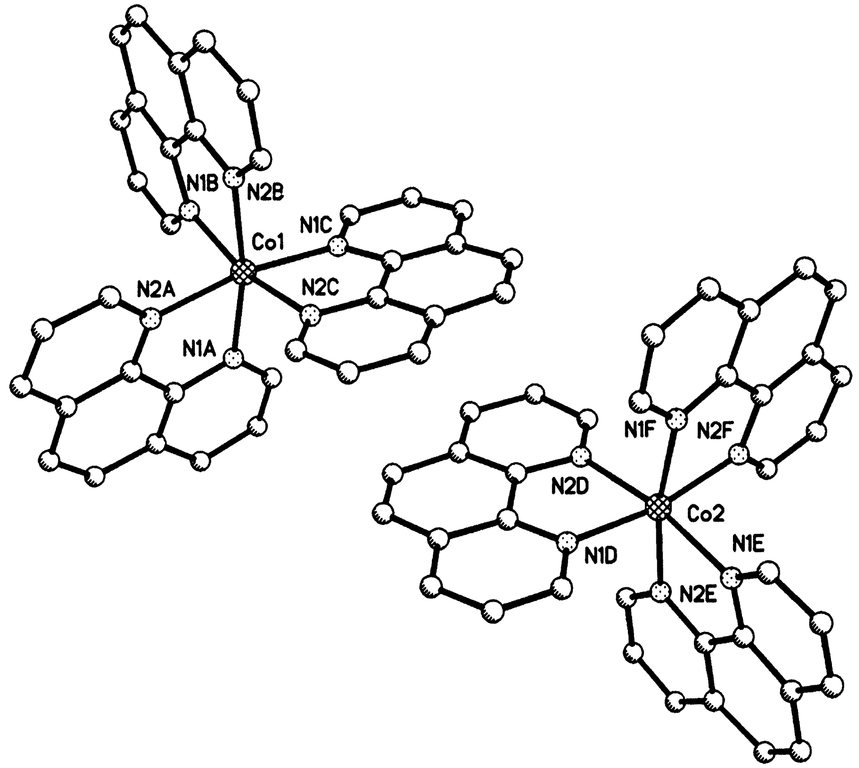

**Figure 3.** Structure of the $[Co(phen)_3]^{2+}$ moieties in $\{[Co(phen)_3](bdoa)\}_2\cdot 24H_2O$ (**2b**) (hygrogen atoms omitted).

The bdoa complexes, the metal-free ligands and the simple acetate complexes of $Co^{2+}$, $Mn^{2+}$ and $Cu^{2+}$ were each tested for their ability to inhibit the growth of three clinical isolates of *C. albicans* (Table 4). Although uncomplexed $bdoaH_2$ is essentially inactive against all three isolates coordination to $Co^{2+}$and $Mn^{2+}$ (complexes **1a** and **5**) leads to slightly increased activity, with the $Co^{2+}$ complex being marginally the better. The equivalent $Cu^{2+}$ complex **6** was inactive. The simple monocarboxylate salt $Co(O_2CCH_3)_2\cdot 4H_2O$ showed moderate anti-*Candida* activity against isolate 1 and isolate 3 but was relatively inactive against isolate 2. $Mn(O_2CCH_3)_2{\cdot}4H_2O$ and $[Cu_2(O_2CCH_3)_4(H_2O)_2]$ were essentially inactive against the three *Candida* isolates.

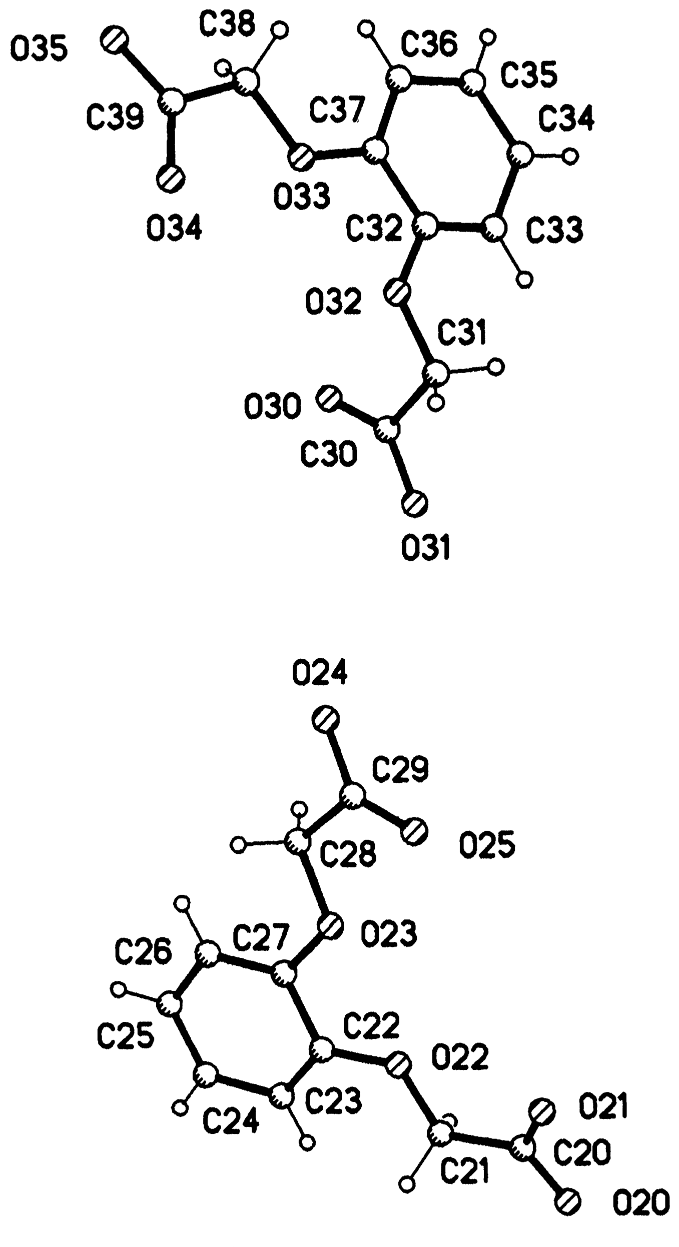

**Figure 4.** Structure of the bdoa$^{2-}$ counterions in $\{[Co(phen)_3](bdoa)\}_2{\cdot}24H_2O$ (**2b**).

The $Co^{2+}$, $Mn^{2+}$ and $Cu^{2+}$ 1,10-phenanthroline adducts **2a**, **3** and **7** dramatically inhibit the growth of all isolates, with the $Mn^{2+}$ and $Cu^{2+}$ species being the more superior. Furthermore, the $Mn^{2+}$ complex **3** is significantly more active than its $Cu^{2+}$ analogue **7** against isolate 2. Thus, it is evident that complexation of 1,10-phenanthroline to the metal centres in the conversion of the $Mn^{2+}$ and $Cu^{2+}$ species **5** and **6** to **3** and **7**, respectively, greatly enhances their anti-*Candida* activities. In contrast to this improved activity with the $Mn^{2+}$ and $Cu^{2+}$ complexes the $Co^{2+}$ non-phenanthroline and 1,10-phenanthroline chelated complexes (**1a** and **2a**), have very similar activities. "Metal-free" 1,10-phenanthroline showed very high anti-*Candida* activity and, indeed, this was only surpassed by the activity of the $Mn^{2+}$ 1,10-phenanthroline complex **3** (on isolates 2 and 3).

**Table 3.** Selected bond lengths [Å] and angles [°] for {[Co(phen)$_3$](bdoa)}$_2$·24$H_2O$ (**2b**).

| | | | |
|---|---|---|---|
| Co(1)-N(2C) | 2.113(11) | Co(1)-N(1C) | 2.124(11) |
| Co(1)-N(1B) | 2. 131(11) | Co(1)-N(2A) | 2.144(10) |
| Co(1)-N(2B) | 2.145(10) | Co(1)-N(IA) | 2.150(12) |
| Co(2)-N(1D) | 2.125(11) | Co(2)-N(2E) | 2.126(11) |
| Co(2)-N(1F) | 2.126(10) | Co(2)-N(2F) | 2.133(11) |
| Co(2)-N(1E) | 2.144(10) | Co(2)-N(2D) | 2.144(10) |
| N(2C)-Co(1)-N(IC) | 79.1(4) | N(2C)-Co(1)-N(1B) | 167.1(4) |
| N(1C)-Co(1)-N(1B) | 94.6(4) | N(2C)-Co(1)-N(2A) | 95.3(4) |
| N(IC)-Co(1)-N(2A) | 169.7(4) | N(1B)-Co(1)-N(2A) | 92.5(4) |
| N(2C)-Co(1)-N(2B) | 90.8(4) | N(1C)-Co(1)-N(2B) | 93.4(4) |
| N(1B)-Co(1)-N(2B) | 78.3(4) | N(2A)-Co(1)-N(2B) | 95.3(4) |
| N(2C)-Co(1)-N(1A) | 98.6(4) | N(1C)-Co(1)-N(1A) | 94.3(4) |
| N(1B)-Co(1)-N(1A) | 93.0(4) | N(2A)-Co(1)-N(1A) | 77.9(4) |
| N(2B)-Co(1)-N(1A) | 168.8(4) | N(2F)-Co(2)-N(2D) | 93.5(4) |
| N(1E)-Co(2)-N(2D) | 169.9(4) | N(1D)-Co(2)-N(2E) | 94.3(4) |
| N(1D)-Co(2)-N(IF) | 93.8(4) | N(2E)-Co(2)-N(1F) | 169.9(4) |
| N(1D)-Co(2)-N(2F) | 169.1(4) | N(2E)-Co(2)-N(2F) | 94.6(4) |
| N(1F)-Co(2)-N(2F) | 77.9(4) | N(1D)-Co(2)-N(1E) | 95.1(4) |
| N(2E)-Co(2)-N(1E) | 78.3(4) | N(IF)-Co(2)-N(1E) | 95.0(4) |
| N(2F)-Co(2)-N(1E) | 92.7(4) | N(1D)-Co(2)-N(2D) | 79.9(4) |
| N(2E)-Co(2)-N(2D) | 93.3(4) | N(1F)-Co(2)-N(2D) | 94.1(4) |

**Table 4.** Anti-*Candida* activity.[a]

| Test compound | % Cell growth | | |
|---|---|---|---|
| | Isolate 1 | Isolate 2 | Isolate 3 |
| Control | 100 | 100 | 100 |
| bdoaH$_2$ | 91 ± 8 | 105 ± 12 | 103 ± 9 |
| aniline | 86 ± 7 | 84 ± 10 | 68 ± 8 |
| imidazole | 78 ± 5 | 92 ± 8 | 98 ± 9 |
| ammonia | 102 ± 10 | 89 ± 8 | 86 ± 7 |
| pyridine | 104 ± 11 | 95 ± 4 | 83 ± 7 |
| triphenylphosphine | 98 ± 12 | 91 ± 4 | 83 ± 7 |
| 1,10-phenanthroline | 12 ± 0.3 | 14 ±0.5 | 15 ± 2 |
| 2,2'-bipyridine | 75 ± 6 | 80 ± 4 | 104 ± 8 |
| Co(CI$_{.3}$CO$_2$)$_2$·4$H_2O$ | 78 ± 5 | 85 ± 8 | 68 ± 5 |
| Mn(CH$_3$CO$_2$)$_2$·4$H_2O$ | 85 ± 8 | 94 ± 5 | 102 ± 8 |
| [Cu$_2$(CH$_3$CO$_2$)$_4$($H_2O$)$_2$] | 108 ± 9 | 89 ± 5 | 92 ± 7 |
| [Co(bdoa)($H_2O$)$_3$]·$H_2O$ **1a** | 63 ± 6 | 70 ± 3 | 78 ± 6 |
| [Mn(bdoa)($H_2O$)$_3$] **5** | 80 ± 1 | 84 ± 4 | 85 ± 1 |
| [Cu(bdoa)]·2$H_2O$ **6** | 101 ± 6 | 93 ± 6 | 91 ± 10 |
| [Co(phen)$_3$]bdoa·10$H_2O$ **2a** | 55 ± 3 | 65 ± 9 | 77 ± 10 |
| [Mn(bdoa)(phen)$_2$]·$H_2O$ **3** | 10 ± 3 | 6 ± 2 | 7 ± 1 |
| [Cu$_2$(bdoa)(phen)$_4$]bdoa·13$H_2O$ **7** | 15 ± 2 | 22 ± 5 | 11 ± 3 |
| [Cu$_2$(bdoa)(bipy)$_4$]bdoa·8.6$H_2O$ **8** | 96 ± 15 | 87 ± 9 | 97 ± 8 |
| [Cu$_2$(bdoa)(an)$_2$]$_2$an **9** | 91 ± 3 | 88 ± 4 | 98 ± 11 |
| [Cu(bdoa)(imid)$_2$] **4** | 95 ± 9 | 99 ± 9 | 98 ± 6 |
| [Cu(bdoa)(NH$_3$)$_2$]·$H_2O$ **10** | 96 ± 6 | 96 ± 11 | 82 ± 7 |
| [Cu(bdoa)(py)$_2$]·$H_2O$ **11** | 91 ± 5 | 97 ± 5 | 77 ± 7 |
| [Cu(bdoaH)(PPH$_3$)$_3$]·$H_2O$ **12** | 99 ± 1 | 89 ± 5 | 72 ± 6 |
| [Cu(phen)(PPH$_3$)$_2$]bdoaH **13** | 63 ± 10 | 88 ± 11 | 65 ± 7 |

[a]Compounds were tested at a concentration of 20 μg cm$^{-3}$ of aqueous RPMI medium. Complexes **5-7**, **9-13** were insoluble in water and were used as suspensions. Yeast cells were grown for 24 h at 37 °C. Results are presented as % cell growth and the effectiveness of the compounds are compared to the growth of the control (no added compound).

In contrast to the performance of 1,10-phenanthroline the other NN chelating agent used in this study, 2,2'-bipyridine, is inefficient at preventing the growth of *Candida* cells.Also, the $Cu^{2+}$ bipyridine complex **8** is ineffective.

Complexation of the monodentate N donor ligands aniline, imidazole, ammonia and pyridine to $Cu^{2+}$ (complexes **9**, **4**, **10** and **11**, respectively) does not improve the poor anti-*Candida* activity of the respective free ligands. Whereas the tris(triphenylphosphine) $Cu^{+}$ complex **12** is only moderately active against isolate 3 the bis(triphenylphosphine)/phenanthroline $Cu^{+}$ species **13** is relatively active against isolates 1 and 3.

In conclusion, the 1,10-phenathroline molecule, whether it be uncomplexed or chelated to a metal centre, exhibits potent anti-*Candida* activity. It is also apparent that the nature of the metal centre to which the 1,10-phenathroline is coordinated is also of significance given that the $Mn^{2+}$ and $Cu^{2+}$ complexes are superior to $Co^{2+}$ complex. The fact that the related NN chelating ligand 2,2'-bipyridine and its copper complex both show negligible activity against the yeast serves to highlight the fundamental structural and reactivity differences between 2,2'-bipyridine and 1,10-phenanthroline.

**References**

1. T. J. Walsh, J. W. Hiemenz and E. Anaissie, *Infect. Dis. Clin. North Am.*, 1996, **29**, 365.
2. A. H. Groll, *J. Infect.*, 1996, **33**, 23.
3. G. Y. Minamoto and A. S. Rosenberg, *Med. Clin. North Am.*, 1997, **81**, 381.
4. A. H. Groll, A. J. De Lucca and T. J. Walsh, *Trends in Microbiology,* 1998, **6**, 117.
5. For example: R. C. Sharma, J. Ambwani, and V. K. Varshney, *J. Indian Chem. Soc.*, 1992, **69**, 770; R. C. Sharma, and R.K. Parashar, *J. Inorg. Biochem.*, 1988, **32**,163.
6. E. A. Green, W. L. Duax, G. M. Smith and F. Wudl, *J. Am. Chem. Soc.* 1975, **97**, 6689.
7. G. Smith, E. J. O'Reilly and C. H. L. Kennard, *Polyhedron*, 1987, **6**, 871.
8. H. B. Kerfoot, G. R. Choppin and T. J. Kistenmacher, *Inorg. Chem.*, 1979, **18**, 787.
9. M. McCann, M. T. Casey, M. Devereux, M. Curran, C. Cardin and A. Todd, *Polyhedron*, 1996, **15**, 2117.
10. M. McCann, J. F. Cronin, M. Devereux, V. McKee and G. Ferguson, *Polyhedron*, 1995, **14**, 3617.
11. M. McCann, M. Devereux, C. Cardin and M. Convery, *Polyhedron*, 1994, **13**, 221.
12. M. Devereux, M. McCann, J. F. Cronin, C. Cardin, M. Convery and V. Quillet, *Polyhedron*, 1994, **13**, 2359.
13. F. Cronin, Ph.D. Thesis, National University of Ireland Maynooth, 1996.
14. G.M. Sheldrick, SHELX97, Universität Göttingen, Germany, 1997.
15. NCCLS (National Committee for Clinical Laboratory), Reference method for broth dilution antifungal susceptibility testing of yeasts; proposed standard. NCCLS, Villanova, Pa. NCCLS publication (1979) M27-P.
16. Although the unit cell dimensions of **1b** have previously been published (see reference 7) the full X-ray crystal structure data were not reported.

**Received: January 4, 1999 - Accepted: January 29, 1999 - Received in revised camera-ready format: January 30, 1999**

